# Case report: A report of the complete pathological response of intrahepatic cholangiocarcinoma after conversion therapy

**DOI:** 10.3389/fimmu.2022.1051130

**Published:** 2022-12-23

**Authors:** Xi Zhang, Hao Tang, Jun Fan, Rui Wang, Yunwei Han, Song Su, Yu Gan, Fangyi Peng, Mingyue Rao, Jianwen Zhang, Bo Li, Xiaoli Yang

**Affiliations:** ^1^ Department of General Surgery (Hepatobiliary Surgery), The Affiliated Hospital of Southwest Medical University, Luzhou, China; ^2^ Academician (Expert) Workstation of Sichuan Province, The Affiliated Hospital of Southwest Medical University, Luzhou, China; ^3^ Department of Oncology, The Affiliated Hospital of Southwest Medical University, Luzhou, China

**Keywords:** conversion therapy, cholangiocarcinoma, case report, gemcitabine, cisplatin, carrelizumab, albumin paclitaxel

## Abstract

Cholangiocarcinoma (CCA) is a rare disease with poor prognosis, and surgery remains the only curative treatment option. However, surgery is inappropriate for the majority of patients with CCA. Conversion therapy may provide opportunities for the surgical treatment of these patients. Herein, we describe a patient with intrahepatic CCA who was first treated with albumin-bound paclitaxel, cisplatin, and gemcitabine in combination with camrelizumab. The patient then successfully underwent surgery and achieved pathological complete remission. This report can serve as a reference for clinicians regarding conversion therapy for intrahepatic CCA.

## Introduction

CCA is an extremely aggressive cancer. It now accounts for approximately 15% of all primary liver cancers and 3% of all gastrointestinal malignancies (1), and its incidence has been rising globally. Indeed, there has been a surge in CCA incidence and related mortality in recent decades (2). CCA has an extremely poor prognosis. The average 5-year overall survival of patients with early-stage CCA who undergo resection is reported to be low at 13%–21% (3, 4). Most patients present with advanced CCA that cannot be surgically resected at diagnosis (5). Few treatment choices exist for such patients. The median overall survival of patients who receive the usual first-line chemotherapy with gemcitabine and cisplatin is believed to be <1 year (6).

Antibodies against programmed cell death-1 (PD-1) and programmed cell death ligand-1 (PDL1) are immune checkpoint inhibitors that have had tremendous success in treating several malignancies. In multiple ongoing clinical trials (NCT03895970, NCT04361331, NCT04454905, NCT03486678, and NCT03779100), the combination of immunotherapy and targeted medications is being evaluated as a possible treatment option for CCA.

Numerous cancer types have recently been studied with regard to the combination of immunotherapy and chemotherapy, and the results have revealed promising antitumor activity. SHR-1210 (camrelizumab) combined with gemcitabine plus cisplatin showed promising safety and efficacy in recurrent or metastatic nasopharyngeal carcinoma (7). Moreover, camrelizumab + GEMOX demonstrated encouraging anticancer activity and a tolerable safety profile as first-line therapy in patients with advanced biliary tract cancer in a single-arm, open-label, phase II trial (NCT03486678) (8). Conversion therapy is defined as the lowering of unresectable tumor volume *via* interdisciplinary systematic treatment to render the tumor resectable. Conversion therapy is widely used in hepatocellular carcinoma, and complete pathological necrosis can be achieved after conversion therapy. In literature, only few cases have been reported on the complete pathological necrosis of CCA after conversion therapy. Herein, we report a case of successful conversion therapy with complete pathological necrosis following treatment with cisplatin, albumin-bound paclitaxel, and gemcitabine in combination with camrelizumab.

## Case description

**Figure 1:**
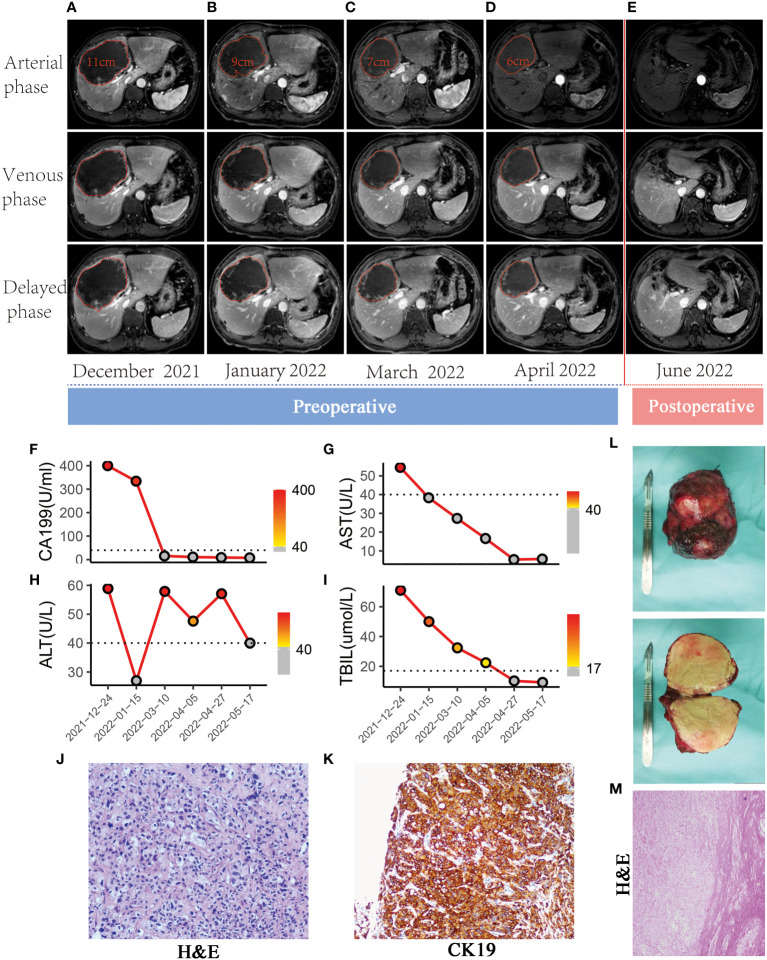
Imaging, laboratory tests, and pathology of the patient. **(A–E)** After systemic chemotherapy, the tumor size was reduced from 11 to 6 cm, and the patient was then treated with surgery. Postoperative imaging revealed no tumor lesions. **(F)** CA199 level decreased from 400 U/mL to 15 U/mL and remained normal thereafter. **(G–I)** The liver function damage could be controlled. AST, aspartate aminotransferase; DBIL, direct bilirubin; TBIL, total bilirubin. **(J)** H&E staining of the biopsy tissue (400× magnification) showed poorly differentiated cholangiocarcinoma. **(K)** Immunohistochemistry revealed strong CK19 expression, suggesting cholangiocarcinoma. **(L)** Macroscopic findings of the resected tumor (size: 7 × 7 × 6 cm). **(M)** H&E staining of the resected tissue (400× magnification) suggested poorly differentiated cholangiocarcinoma and pathological necrosis of the tumor. No microvascular invasion was observed.

A 36-year-old man who had been experiencing epigastric distention for >1 month visited our hospital. He had no prior history of hepatitis B or C infection or any other illnesses, and the Child–Pugh score of the patient was 7 (class B). Contrast-enhanced abdominal magnetic resonance imaging (MRI) revealed a 110 × 108-mm lesion in the liver with enlarged hilar lymph nodes (**Figure 1A**). Chest computed tomography and emission computed tomography did not reveal significant tumor metastasis. Before initiating treatment, the patient’s CA199 level was >400 U/mL (**Figure 1F**). Needle biopsy revealed a heterotypic cell mass (hematoxylin and eosin [H&E] staining, 400× magnification), which was strongly positive for cytokeratin (CK)19 on immunohistochemistry (**Figures 1J, K**). There is no clear causal relationship between the expression of PD1 and PDL1 and the prognosis of patients in the current treatment of cholangiocarcinoma. The relationship between TMB and BTC is controversial and remains to be further clarified, and available evidence seems to suggest an overall modest value of these biomarkers. For all these reasons, we believe that testing the patient’s PDL1, TMB, and dMMR/MSI is currently of little relevance, so we did not do these tests. The expression of PD-1 and PD-L1, tumor mutation burden, and microsatellite instability status were not evaluated. The tumor was considered CCA (T2N1M0, IIIB) according to the 8^th^ American Joint Committee on Cancer tumor–node–metastasis staging system for CCA. After discussion with the medical team, the patient was administered five three-week cycles of chemotherapy (gemcitabine, 1000 mg/m^2^ on days 1 and 8; cisplatin, 25 mg/m^2^ on days 1 and 8, and albumin-bound paclitaxel, 125 mg/m^2^ on days 1 and 8; the patient also received 200 mg camrelizumab on day 1 for five three-week cycles). The first systemic therapy was administered in December 2021, the second in January 2022, the third in March 2022, and the fourth and fifth in April 2022. After each cycle there was a noticeable reduction in the tumor size (**Figures 1A–D**). After the fifth cycle of treatment, contrast-enhanced MRI showed little enhancement and few enlarged lymph nodes (**Figures 1B–D**). CA199 level reduced significantly after each treatment and remained normal at the end of chemotherapy (**Figure 1F**). Moreover, no fever, rash, myelosuppression, or other side effects were observed other than moderate liver damage (**Figures 1G–I**). A three-dimensional reconstruction of postchemotherapy computed tomography images revealed that the volume of the liver was 1642.08 mL and that of the lesion was 262.61 mL. The patient underwent surgical resection in May 2022, and during surgery, the tumor (approximately 7 × 7 × 6 cm) was found in the right lobe of the liver (**Figure 1L**). Postoperative pathological examination revealed poorly differentiated CCA, with no satellite lesion or portal vein tumor thrombus. The examination also showed pathological complete necrosis of the tumor, and no lymph node metastasis or microvascular invasion was observed (**Figure 1M**). Moreover, no postoperative complications (such as biliary leakage) were observed, and owing to good wound healing, the patient was discharged a week later. Surgical resection was followed by one cycle of systemic therapy. (gemcitabine, 1000 mg/m^2^ on days 1 and 8; cisplatin, 25 mg/m^2^ on days 1 and 8, and albumin-bound paclitaxel, 125 mg/m^2^ on days 1 and 8; the patient also received 200 mg camrelizumab on day 1 for five three-week cycles). Follow-up MRI (before the next cycle of systemic therapy) performed 1 month after surgery revealed no recurrence (**Figure 1E**). To maintain remission, carelizumab will continue to be used every three weeks for one year.

## Discussion

Because CCAs are aggressive tumors, the majority of patients are diagnosed at advanced stages (9). Regardless of the anatomical subtype of this cancer, the current first-line chemotherapy for patients with advanced-stage CCA not susceptible to locoregional and surgical alternatives is the combination of gemcitabine and cisplatin (10). Shroff et al. (11) studied 60 patients with advanced biliary tract cancer who underwent treatment with nab-paclitaxel plus gemcitabine-cisplatin; 38 (63%) patients had intrahepatic CCA. With respect to these patients, the median follow-up was 12.2 months, the median progression-free survival was 11.8 months, and the partial response rate was 45%. The study results revealed that the combination of cisplatin, gemcitabine, and albumin-bound paclitaxel extended overall survival compared with only cisplatin and gemcitabine (11). As per the guidelines for the diagnosis and treatment of biliary tract malignancies published in Chinese Society of Clinical Oncology 2021, intense chemotherapy combining three drugs can be considered for patients with good physical condition. For the present patient, camrelizumab was chosen, and after one treatment cycle, re-examination revealed tumor shrinkage. There were no obvious side effects, and the patient chose to continue treatment. After five cycles, the tumor shrank significantly. Thus, the patient underwent surgery. The H&E staining of the resected tumor tissue suggested poorly differentiated CCA and pathological complete necrosis of the tumor, without any lymph node metastasis. This suggests that conversion therapy using three-drug intensive chemotherapy combined with PD-1 is indeed feasible for CCA in patients with good physical condition. In a Phase II trial including 54 patients with biliary tract cancer, nivolumab, a PD-1 inhibitor, achieved an objective response rate of 22% (12). Taken together, the present patient with CCA was treated with intensive chemotherapy, which exhibited a significant effect. This effect may be attributed to the patient’s young age and good physical condition. However, there are some issues worth considering, such as the suitability of this treatment plan with respect to the patient type and whether postoperative adjuvant therapy is needed. In any event, the present report can be used as a reference for conversion therapy in CCA.

## Data availability statement

The original contributions presented in the study are included in the article/supplementary material. Further inquiries can be directed to the corresponding authors.

## Ethics statement

Written informed consent was obtained from the individual(s) for the publication of any potentially identifiable images or data included in this article.

## Author contributions

XY and BL conceived the study. XZ drafted the article and contributed to editing and revision. HT contributed to the editing and revision of the article. JF and RW collected and analyzed literature. FP, YG, and SS assisted with literature review and manuscript preparation. YH, WZ, and YR assisted with literature review and image analysis. All authors contributed to the article and approved the submitted version.

## References

[1] Banales JM Marin JJG Lamarca A Rodrigues PM. Cholangiocarcinoma 2020: the next horizon in mechanisms and management. Nat Rev Gastroenterol Hepatol (2020) 17(9):557–88. doi: 10.1038/s41575-020-0310-z PMC7447603 32606456

[2] Raoof M Singh G. Rising trends in intrahepatic cholangiocarcinoma incidence and mortality: getting at the root cause. Hepatobiliary Surg Nutr (2019) 8(3):301–3. doi: 10.21037/hbsn.2019.01.15 PMC6561886 31245420

[3] Ribero D Pinna AD Guglielmi A Ponti A Nuzzo G Giulini SM. Surgical approach for long-term survival of patients with intrahepatic cholangiocarcinoma: a multi-institutional analysis of 434 patients. Arch Surg (2012) 147(12):1107–13. doi: 10.1001/archsurg.2012.1962 22910846

[4] Xing KL Lu LH Huang X He CB Song YD Guo RP. A novel prognostic nomogram for patients with recurrence of intrahepatic cholangiocarcinoma after initial surgery. Front Oncol (2020) 10:434. doi: 10.3389/fonc.2020.00434 32300559 PMC7142225

[5] Everhart JE Ruhl CE. Burden of digestive diseases in the united states part III: liver, biliary tract, and pancreas. Gastroenterology (2009) 136:1134–44. doi: 10.1053/j.gastro.2009.02.038 19245868

[6] Valle J Wasan H Palmer DH Cunningham D Anthoney A Maraveyas A. Cisplatin plus gemcitabine versus gemcitabine for biliary tract cancer. N Engl J Med (2010) 362(14):1273–81. doi: 10.1056/NEJMoa0908721 20375404

[7] Gandhi L Rodríguez-Abreu D Gadgeel Esteban E Felip E De Angelis F. Pembrolizumab plus chemotherapy in metastatic non-small-cell lung cancer. N Engl J Med (2018) 378:2078–92. doi: 10.1056/NEJMoa1801005 29658856

[8] Chen X Wu X Wu H Gu Y Shao Y Shao Q. Camrelizumab plus gemcitabine and oxaliplatin (GEMOX) in patients with advanced biliary tract cancer: A single-arm, open-label, phase II trial. J Immunother Cancer (2020) 8(2):e001240. doi: 10.1136/jitc-2020-001240 33172881 PMC7656907

[9] Jarnagin WR Fong Y DeMatteo RP Gonen M Burke EC Bodniewicz BS J. Staging, resectability, and outcome in 225 patients with hilar cholangiocarcinoma. Ann Surg (2001) 234:507–17. doi: 10.1097/00000658-200110000-00010 PMC1422074 11573044

[10] Valle J Wasan H Palmer DH Cunningham D Anthoney A Maraveyas A. Cisplatin plus gemcitabine versus gemcitabine for biliary tract cancer. N Engl J Med (2010) 362:1273–81. doi: 10.1056/NEJMoa0908721 20375404

[11] Shroff RT Javle MM Xiao L Bekaii-Saab TS Borad MJ. Gemcitabine, cisplatin, and nab-paclitaxel for the treatment of advanced biliary tract cancers: A phase 2 clinical trial. JAMA Oncol (2019) 5(6):824–30. doi: 10.1001/jamaoncol.2019.0270 PMC6567834 30998813

[12] Kim RD Chung V Alese OB El-Rayes BF Li D Al-Toubah TE. A phase 2 multi-institutional study of nivolumab for patients with advanced refractory biliary tract cancer. JAMA Oncol (2020) 6(6):888–94. doi: 10.1001/jamaoncol.2020.0930 PMC7193528 32352498

